# Sleep Disturbances, Metabolic Markers, and Outcomes After Stroke: A Retrospective Cohort Study in a Tertiary Hospital

**DOI:** 10.3390/jcm15145394

**Published:** 2026-07-09

**Authors:** Fahad Alkhamis, Majed M. Alabdali, Danah Aljaafari, Rudaynah A. Alali, Alawi H. Habara, Mohammed S. Akhtar, Shamim S. Mohiuddin, Hazim H. Habarah, Moyad M. Almuslim, Chittibabu Vatte, Brendan J. Keating, Chan Wang, Amein K. Al-Ali

**Affiliations:** 1 Department of Neurology, King Fahd Hospital of the University, Imam Abdulrahman Bin Faisal University, Dammam 31441, Saudi Arabia; fkhamis@iau.edu.sa (F.A.);; 2 Department of Internal Medicine, King Fahd Hospital of the University, Imam Abdulrahman Bin Faisal University, Dammam 31441, Saudi Arabia; raali@iau.edu.sa; 3 Department of Clinical Biochemistry, College of Medicine, Imam Abdulrahman Bin Faisal University, Dammam 31441, Saudi Arabia; ahhabara@iau.edu.sa (A.H.H.); cbvatte@iau.edu.sa (C.V.); 4 Department of Pathology, King Fahd Hospital of the University, Imam Abdulrahman Bin Faisal University, Dammam 31441, Saudi Arabia; 2250600037@iau.edu.sa; 5 College of Pharmacy, Imam Abdulrahman Bin Faisal University, Dammam 31441, Saudi Arabia; 6 Division of Transplantation, Department of Surgery, New York University Langone Health, New York, NY 10016, USA

**Keywords:** stroke, sleep apnea, insomnia, ischemic, Saudi Arabia

## Abstract

**Background:** Stroke remains a leading cause of death and long-term disability worldwide. Sleep disturbances are increasingly recognized as potential factors influencing recovery after stroke. Therefore, in this study we examined the associations of sleep disturbances and metabolic markers with post-stroke outcomes. **Methods:** We conducted a retrospective study of adult patients with stroke-related presentations. The primary analysis included 270 patients with follow-up mRS data. Extracted variables included demographics, vascular risk factors, stroke subtype, imaging findings, sleep features, and selected laboratory markers. Functional outcome was classified as favorable, mRS 0–2, or unfavorable, mRS 3–6. Recurrent stroke burden was analyzed as 0–1 versus ≥2 documented events. Associations and predictive performance were assessed using group comparisons, LASSO logistic regression, and random forest models with repeated 10-fold cross-validation. **Results:** Among 270 stroke patients, 214 (79.3%) had favorable outcomes and 56 (20.7%) had unfavorable outcomes. Sleep disturbances were common, especially nocturnal awakenings (59.6%), increased sleep apnea risk (44.4%), circadian rhythm disturbances (28.9%), and insomnia (23.7%). Unfavorable outcomes were linked to older age, cardio-aortic embolism, large vessel/cortical stroke, abnormal vascular imaging, insomnia, and lower HDL. In LASSO analysis, age, steno-occlusive/atherosclerotic imaging, cardio-aortic embolism, and insomnia predicted unfavorable outcome, while HDL was protective. For recurrent stroke, small artery occlusion and hypertension with diabetes were retained. In predictive modeling, the best random forest model showed good discrimination AUC values of 0.791 ± 0.0126. **Conclusions:** Poorer stroke outcomes were associated with vascular factors, insomnia, and low HDL; recurrent events were mainly associated with small artery occlusion.

## 1. Introduction

Stroke remains a leading cause of mortality and long-term disability worldwide, affecting millions and placing a significant burden on patients, families, and healthcare systems [1,2]. Despite notable advances in acute stroke care that have improved survival in recent years, many patients continue to experience lasting functional impairments and are at risk of recurrent cerebrovascular events [2]. Recent global epidemiological analyses also emphasize persistent disparities in stroke burden across regions and healthcare systems, underscoring the need for context-specific data from underrepresented populations, including the Middle East [3]. Identifying factors that contribute to poor recovery has become increasingly important for improving prognostic assessments and for guiding post-stroke care strategies.

Several clinical and vascular risk factors, including older age, hypertension, diabetes mellitus, and stroke severity, have been consistently linked to stroke occurrence and poorer outcomes [3,4,5]. In addition to these established predictors, recent investigations, including that of Baylan et al. (2020), highlight that sleep disturbances may play an important role in post-stroke recovery [6,7]. Sleep problems such as insomnia symptoms, prolonged sleep latency, fragmented sleep, circadian rhythm disruption, and sleep apnea are common after stroke and may adversely affect neurological recovery through autonomic dysregulation, impaired metabolic control, inflammation, and reduced neuroplasticity [7,8].

While the association between sleep disorders and cardiovascular risk is increasingly acknowledged, the recognition and management of sleep disturbances in stroke care remain suboptimal. Despite increased awareness, practical challenges such as limited screening protocols and a lack of standardized treatment pathways often hinder effective identification and management of sleep disturbances in stroke patients. For instance, sleep-disordered breathing, particularly obstructive sleep apnea, has been reported in up to 70% of stroke patients, yet remains underdiagnosed and undertreated in routine clinical practice [7,9]. Emerging research indicates that targeted interventions for sleep disorders may improve neurological outcomes, reduce the risk of recurrence, and enhance quality of life for stroke survivors [10,11]. Furthermore, sleep quality has been linked to key metabolic markers, including glucose regulation, lipid profiles, and inflammatory mediators, which are themselves predictors of vascular risk and recovery trajectory after stroke [12,13,14].

There are several ways in which sleep problems might influence how well someone recovers after a stroke. Conditions like insomnia, restless or broken sleep, and sleep-disordered breathing can put extra stress on the body, raising sympathetic activity, hormone levels, and inflammation and even harming blood vessels. These changes may also disrupt how the body manages sugar and fats, such as cholesterol and triglycerides, making it harder for blood vessels to repair themselves and increasing the risk of another stroke or poor recovery [15]. Trouble sleeping may also slow down brain recovery, affect thinking, make rehabilitation more difficult, and interfere with the brain’s waste-clearing system. While our retrospective study cannot directly test these biological pathways, they support the idea of studying sleep symptoms alongside lab measures like HDL, LDL, total cholesterol, triglycerides, and glucose [16,17].

Despite increasing interest in the relationship between sleep and stroke, limited data are available from Saudi and Middle Eastern tertiary-care settings regarding the prognostic relevance of routinely documented sleep-related symptoms after stroke. Also, very few studies in real-life hospital settings have looked at both sleep issues and indicators like cholesterol or blood vessel health to see how they affect recovery or the chance of another stroke. What makes our study unique is that it uses routine hospital data from a Saudi tertiary hospital, and when needed, obtains further details from patients or their caregivers. This allows us to look at how sleep problems, lab markers, and blood vessel health are linked to stroke outcomes. We hypothesized that people with insomnia or a high risk of sleep apnea would have a harder time recovering from stroke, even after accounting for differences in blood-vessel and metabolic health. We also wanted to see whether lab measures like HDL cholesterol were linked to recovery, and whether adding information about sleep and metabolism to regular hospital data could make it easier to predict who might experience worse outcomes. Finally, we looked for factors—both medical and sleep-related—that might be connected to experiencing another stroke.

In this retrospective study, we analyzed medical records from 270 adult patients with ischemic or hemorrhagic stroke admitted to a tertiary hospital. We aimed to evaluate the prevalence of post-stroke sleep disturbances and to investigate whether clinical factors, sleep-related features, and metabolic markers were associated with functional outcomes at follow-up and recurrent stroke events. By integrating routinely collected clinical and laboratory data with sleep-related parameters, our goal was to provide a more nuanced understanding of recovery trajectories and highlight potential avenues for intervention in post-stroke care.

## 2. Materials and Methods

### 2.1. Population, Study Design, and Setting

This was a retrospective cohort study based primarily on medical-record review, supplemented by direct or telephone verification of selected sleep-related and follow-up information when documentation was incomplete. Interviews or telephone calls were conducted after the index stroke-related admission among patients who remained in follow-up or could be contacted. These contacts were used only to verify available clinical and sleep-related information and did not constitute a prospective intervention or standardized sleep assessment protocol.

We analyzed medical records from 270 adult patients with ischemic or hemorrhagic stroke who were admitted to King Fahd Hospital of the University (KFHU), a tertiary care center in the Eastern Province of Saudi Arabia. We included adults who had suffered either an ischemic stroke, hemorrhagic stroke, or a transient ischemic attack between January 2019 and December 2024. Patients were eligible whether it was their first stroke or a recurring event, as long as there was sufficient clinical information available, such as details on blood vessels, metabolism, sleep, and recovery outcomes. The specific type of stroke was determined using the physician’s diagnosis, results from brain scans, discharge summaries, and other vascular tests. If key outcome data were missing, or if we could not find sufficient information to classify the main exposure or outcomes, those patients were not included in the analysis. People who had trouble speaking, were confused, had memory issues, or could not answer phone calls were not automatically excluded, provided we could obtain reliable information from their records or a caregiver. However, if we could not obtain sufficient sleep or follow-up data, those patients were not included in the parts of the study that required such data.

Because our inclusion criteria depended on how complete the medical records and follow-up data were, there is a possibility that our sample does not fully represent all stroke patients. For this reason, our study group should be seen as a real-world sample from a single tertiary hospital, rather than a broad population-based stroke cohort.

### 2.2. Data Collection and Variables

We collected information about sleep issues like insomnia, waking up during the night, changes in sleep patterns, how long it took to fall asleep, and signs of high sleep apnea risk from regular medical notes. When records were incomplete, we asked patients’ or their caregivers by phone to fill in the gaps. It is important to note that these sleep details were not gathered using formal sleep questionnaires, sleep studies, or wearable devices. That means that our sleep measures reflect what was recorded in the clinic or reported by the patient, not diagnoses confirmed with objective tests.

For this study, we defined insomnia as any note or report describing trouble falling asleep, staying asleep, waking up too early, or feeling unrested after sleep. Sleep latency was simply the time it took a patient to fall asleep, according to their own or their caregiver’s accounts. Nocturnal awakenings referred to repeated waking during the night. Circadian rhythm disturbance was defined as documentation or report of an irregular sleep–wake schedule, delayed sleep timing, or reversal of day–night sleep pattern. We also recorded how often and for how long people reported napping during the day. Signs of high sleep apnea risk included things like snoring, observed pauses in breathing, excessive sleepiness during the day, or if a doctor had noted possible obstructive sleep apnea. Since we did not routinely use formal tools like the Insomnia Severity Index or the STOP-Bang questionnaire, all these variables were treated as either documented by clinicians or reported by patients, not as official medical diagnoses.

Extracted information included demographic details, vascular risk factors, stroke subtype, neuroimaging findings, and relevant laboratory results. To address gaps in the documentation of sleep disorders, additional sleep-related data were obtained through direct patient interviews or telephone follow-up when possible. Sleep variables encompassed insomnia symptoms, sleep latency, frequency of nocturnal awakenings, circadian rhythm disturbances, frequency and duration of daytime naps, and risk for sleep apnea. Laboratory measures comprised total cholesterol, HDL and LDL cholesterol, triglycerides, and blood glucose levels. The total number of stroke events experienced by each patient was also recorded.

### 2.3. Outcome Measures

The primary outcome was functional status at follow-up, categorized as favorable (mRS 0–2) or unfavorable (mRS 3–6). The recurrent stroke variable represented the total number of documented stroke events available in the medical record. A value of 0 indicated a stroke-related presentation without a confirmed documented stroke event in the available record, whereas values of 1 or more indicated documented stroke events. Because event dates and uniform follow-up durations were not consistently available, time-to-event or recurrent-event modeling was not performed. The recurrent-event analysis was therefore considered exploratory.

For follow-up data, we checked each patient’s most recent functional status after leaving the hospital, using either the clinic records or, if needed, information verified by phone. Because follow-up appointments happened as part of regular medical care and not based on a set research schedule, the time between hospital discharge and follow-up was not the same for everyone. We recognize this as a limitation in our study. Deaths during the follow-up period were assigned an mRS score of 6 and were included in the unfavorable outcome group. Patients who did not have any follow-up mRS data were not included in the main outcome analysis. The follow-up mRS score came from the last available clinic note or, if needed, a phone verification. Since these assessments did not always happen at specific time-points, such as 30, 90, or 180 days, differences in follow-up length could have affected how we classified outcomes, and we acknowledge this as another limitation.

The secondary outcome was the number of stroke events per patient documented in the medical record. The distribution of stroke event counts was highly skewed, with over 90% of patients having a single documented event, only a small proportion experiencing multiple events, and one patient having no documented event. Accordingly, this variable was dichotomized as 0–1 documented event versus recurrent events (≥2 events) for subsequent analyses.

### 2.4. Statistical Analysis

In the univariate analysis, baseline characteristics were summarized as mean (SD) for continuous variables and as counts (percentages) for categorical variables. Comparisons between binary outcome groups were performed using chi-square tests [18] for categorical variables and *t*-tests [19] for continuous variables. Statistical significance was defined as a two-sided *p*-value < 0.05. All analyses were observational and exploratory. Statistical associations were interpreted as evidence of relationships between variables and outcomes, not as evidence of causality. No causal inference methods were applied, and residual confounding from measured and unmeasured factors remains possible.

### 2.5. Missing-Value Handling

EEG/ECG and glucose variables were excluded from the multivariate analyses because of their high proportions of missing data (>40%). The retained predictors were age, sex, final diagnosis, territory of infarction, vascular imaging findings, hemorrhage, cardio-aortic embolism, large artery atherosclerosis, small artery occlusion, undetermined causes, uncommon causes, insomnia, circadian rhythm disturbances, nocturnal awakenings, sleep apnea risk, sleep latency, nap frequency, nap duration, sub-clinical status, drug administered, HDL cholesterol, LDL cholesterol, total cholesterol, and triglycerides. Remaining missing data were handled using multiple imputation by chained equations in the R package mice (mice version 3.16.0) [20], with predictive mean matching for continuous variables, logistic regression for binary variables, and polytomous logistic regression for categorical variables with more than two levels. Because of the retrospective nature of the study and the limited number of recurrent events, sensitivity analyses were interpreted cautiously. The lack of extensive sensitivity analyses is acknowledged as a limitation.

### 2.6. Penalized Regression Analysis

We used penalized logistic regression with the least absolute shrinkage and selection operator (LASSO) penalty [21] to identify predictors of unfavorable functional outcome (mRS 3–6 vs. 0–2), as well as recurrent stroke events (0–1 documented event vs. ≥2 events). Candidate predictors included demographic, clinical, sleep-related, and laboratory variables. The tuning parameter was selected using 10-fold cross-validation (CV), and variables with non-zero coefficients were retained. Bootstrap resampling was used to estimate 95% confidence intervals for the selected coefficients.

### 2.7. Predictive Analysis

A random forest (RF) classifier [22] was used to assess prediction of functional outcome (favorable vs. unfavorable mRS). Model performance was evaluated using 100 repetitions of 10-fold CV. In each repetition, patients were randomly divided into 10 folds; each fold was held out once for testing while the model was trained on the remaining nine folds with repeated CV, and receiver operating characteristic (ROC) analysis [23] was performed on the pooled out-of-fold predictions from all subjects. Predictive performance was summarized by the mean and standard deviation of the area under the ROC curve (AUC) [23]. Three predictor sets were evaluated: all predictors [19,20,21,22,23], a reduced set of top-ranked predictors selected until cumulative relative importance reached 0.8 [23], and a routine clinical set consisting of age, hypertension, and T2D status. Variable importance was quantified by mean decrease in accuracy [24]. All statistical analyses were performed using R (R Core Team, R Foundation of Statistical Computing) version 4.4.3.

In the original analysis, the RF. Top predictor set was selected using variable importance from the full dataset before cross-validation. We recognize that this approach may introduce information leakage and produce optimistic AUC estimates. Therefore, we have revised the Methods and Discussion to describe the random forest analysis as exploratory and hypothesis-generating. Where possible, we repeated the analysis using feature selection within cross-validation folds; otherwise, this limitation is now explicitly acknowledged [25,26].

## 3. Results

### 3.1. Cohort Characteristics

Among 270 patients with available follow-up mRS data, 214 (79.3%) had a favorable outcome and 56 (20.7%) had an unfavorable outcome (Table 1 and Table S1). The full follow-up mRS distribution was as follows: mRS 0, (n = 63) patients [23.33%]; mRS 1, (n = 104) patients [38.52%]; mRS 2, (n = 47) patients [17.41%]; mRS 3, (n = 39) patients [14.44%]; mRS 4, (n = 11) patients [4.07%]; mRS 5, five patients [1.85%]; and mRS 6, (n = 1) patient [0.37%]. Deaths were classified as mRS 6 and included in the unfavorable-outcome group.

Most patients had a final diagnosis of stroke (97.8%), while 2.2% had transient ischemic attacks. The mean number of stroke events per patient was 1.14 ± 0.57. The mean age of the cohort was 54.5 ± 13.1 years, and 60.7% were male. Sleep-related disturbances were frequently documented, including nocturnal awakenings (59.6%), elevated sleep apnea risk (44.4%), circadian rhythm disturbances (28.9%), and insomnia (23.7%). Mean sleep latency was 32.5 ± 16.9 min.

### 3.2. Associations with the Primary Outcome

**Figure 1:**
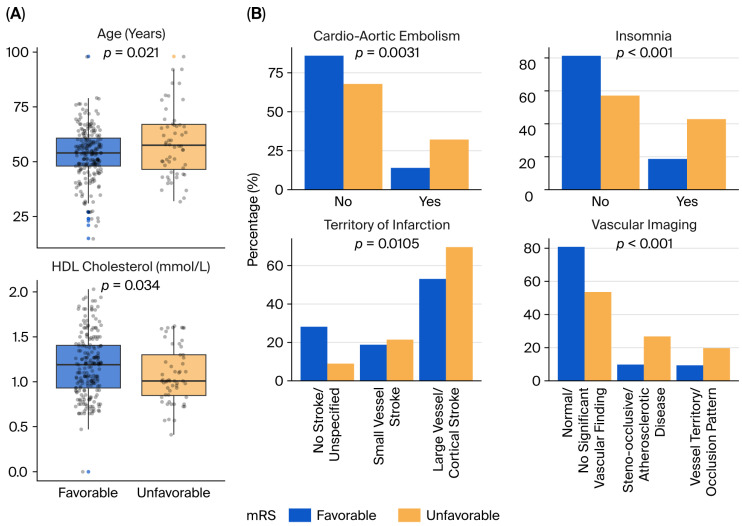
Group comparisons of selected variables according to functional outcome after stroke. (**A**) Boxplots show distributions of age and HDL cholesterol in patients with favorable and unfavorable modified Rankin Scale (mRS) outcomes. *p*-values were calculated using the *t*-test. (**B**) Group comparisons for cardio-aortic embolism, insomnia, territory of infarction, and vascular imaging findings according to mRS category. *p*-values were calculated using the chi-square test.

Compared with patients with favorable outcomes (Table 1 and Figure 1), those with unfavorable functional outcomes were older (58.7 ± 15.6 vs. 53.4 ± 12.1 years, *p* = 0.021) and had lower HDL cholesterol levels (1.07 ± 0.32 vs. 1.18 ± 0.35 mmol/L, *p* = 0.034). They were also more likely to have cardio-aortic embolism (32.1% vs. 14.0%, *p* = 0.003), large vessel/cortical stroke within the infarct territory classification (69.6% vs. 52.8%, *p* = 0.011), and abnormal vascular imaging findings (*p* < 0.001). Among the sleep-related variables, insomnia was more common in the unfavorable-outcome group than in the favorable-outcome group (42.9% vs. 18.7%, *p* < 0.001). Elevated sleep apnea risk was more frequent in the unfavorable-outcome group than in the favorable-outcome group, although this difference did not reach statistical significance (55.4% vs. 41.6%, *p* = 0.090). The remaining independent variables did not differ significantly. The number of stroke events per patient was also similar between groups (*p* = 0.295).

**Figure 2:**
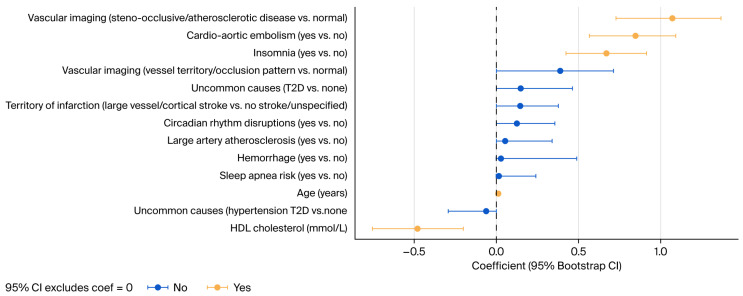
Penalized logistic regression (LASSO) coefficients for predictors of unfavorable functional outcome. Points represent coefficient estimates and horizontal bars indicate 95% bootstrap confidence intervals.

In the LASSO penalized logistic regression analysis, several variables were retained as predictors of unfavorable functional outcome (Figure 2). As expected, age was retained and showed a positive association with unfavorable outcome. In addition, vascular imaging demonstrating steno-occlusive/atherosclerotic disease relative to normal findings, cardio-aortic embolism, and insomnia were also positively associated with unfavorable outcome. In contrast, HDL cholesterol was inversely associated with unfavorable outcome. The corresponding regression coefficients, 95% confidence intervals, odds ratios, and 95% odds-ratio confidence intervals for the retained LASSO predictors are provided in Supplementary Tables S2 and S3.

### 3.3. Associations with the Secondary Outcome

**Figure 3:**
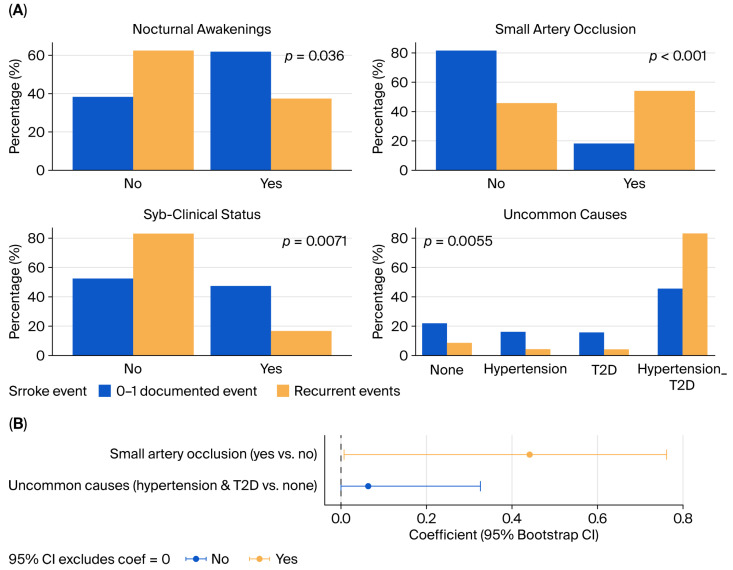
Univariate and penalized regression analyses of recurrent stroke events. (**A**) Group comparison of selected variables according to stroke event burden. Bar plots show the distribution of nocturnal awakenings, small artery occlusion, sub-clinical status, and uncommon causes in patients with 0–1 documented stroke event and those with recurrent events (≥2 events). *p*-values were calculated using the chi-square test. (**B**) Penalized logistic regression (LASSO) coefficients for predictors of recurrent stroke events. Points represent coefficient estimates and horizontal bars indicate 95% bootstrap confidence intervals.

In univariate analyses (Table 2 and Figure 3A, Supplementary Table S2), patients with recurrent stroke events (≥2 events) differed significantly from those with 0–1 documented event in several clinical and sleep-related characteristics. Because only 24 patients had recurrent stroke events, these analyses were considered exploratory and may be underpowered for stable multivariable inference.

Small artery occlusion was more common in the recurrent-event group (54.2% vs. 18.3%, *p* < 0.001), whereas sub-clinical status was less frequent (16.7% vs. 47.6%, *p* = 0.007). The distribution of uncommon causes also differed significantly between groups (*p* = 0.006), with hypertension plus type 2 diabetes being more prevalent among patients with recurrent events (83.3% vs. 45.9%). In addition, nocturnal awakenings were less frequently reported in the recurrent-event group (37.5% vs. 61.8%, *p* = 0.036). Other demographic, stroke-related, sleep-related, and laboratory variables did not differ significantly between groups.

In the LASSO penalized logistic regression analysis, small artery occlusion and hypertension plus type 2 diabetes versus none were retained as predictors of recurrent stroke events (Figure 3B), both with positive coefficients. Small artery occlusion was positively associated with recurrent stroke events, with its 95% confidence interval excluding zero. Hypertension plus type 2 diabetes also showed a positive coefficient, suggesting an increased risk of recurrent stroke events; however, its 95% confidence interval slightly crossed zero, indicating a less stable association.

### 3.4. Prediction of the Primary Outcome

**Figure 4:**
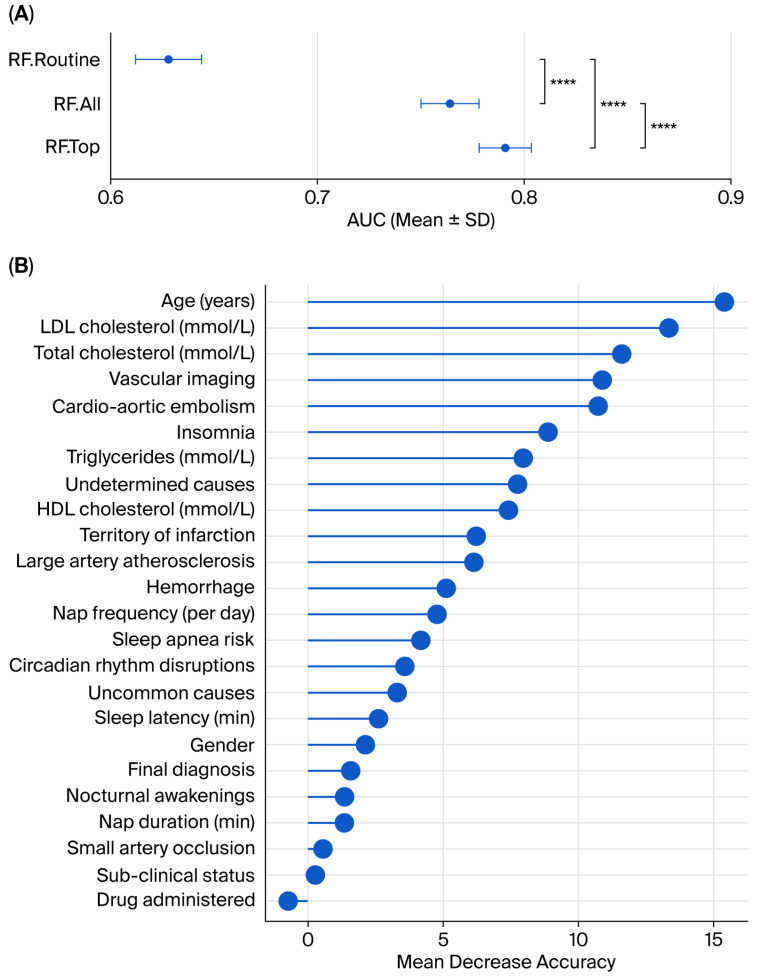
Random forest prediction of functional outcome after stroke. (**A**) Predictive performance of the three random forest models, expressed as mean AUC ± SD across 100 repetitions of 10-fold cross-validation. RF.Top showed the highest performance, followed by RF.All and RF.Routine; all pairwise comparisons were significant (**** adjusted *p* < 0.0001). (**B**) Variable importance ranked by mean decrease in accuracy.

As expected, age emerged as the strongest predictor of functional outcome as assessed using the mRS. However, the routine clinical model, which included only age, hypertension, and T2D status, demonstrated limited discriminatory ability (AUC 0.628 ± 0.0159) (Figure 4).

In contrast, the reduced random forest model based on the top-ranked predictors (RF.Top) achieved the highest predictive performance for functional outcome (AUC 0.791 ± 0.0126), followed by the full model including all predictors (RF.All: AUC 0.764 ± 0.0140). The model including all predictors performed significantly better than the routine clinical model, and the top-ranked predictor model further significantly outperformed both the full model and the routine model (all adjusted *p* < 0.0001). In addition to age, variable importance analysis identified LDL cholesterol, total cholesterol, vascular imaging findings, cardio-aortic embolism, and insomnia as among the most influential predictors.

## 4. Discussion

This retrospective study from King Fahd University Hospital in Saudi Arabia explored the associations between clinical, vascular, metabolic, and sleep-related factors with functional recovery and recurrent stroke in a real-world hospital cohort. Patients with documented insomnia, abnormal vascular imaging, cardio-aortic embolism, older age, and lower HDL cholesterol were more likely to have unfavorable functional outcomes, while small artery occlusion and the combined presence of hypertension and type 2 diabetes were associated with recurrent stroke burden. Because of the retrospective and observational design, these findings should be interpreted as associations rather than evidence of direct causality. Our cohort was characterized by a mean age of 54.5 years and a predominance of male patients, mirroring the relatively younger age at stroke onset that is frequently seen in Gulf and Middle Eastern populations compared to Western cohorts [1,2]. In our cohort, this earlier onset may reflect the substantial burden of vascular risk factors, such as hypertension and type 2 diabetes, that we observed in almost half of our patients [3]. Our analysis found that older age, abnormal vascular imaging, cardio-aortic embolism, large vessel/cortical stroke, lower HDL cholesterol, and insomnia were associated with unfavorable functional outcomes at follow-up. While these findings are consistent with international reports, their relevance in our setting supports further investigation of risk-factor management strategies tailored to Saudi patients [4,5,27]. Our cohort was characterized by a mean age of 54.5 years and a predominance of male patients, mirroring the relatively younger age at stroke onset that is frequently seen in Gulf and Middle Eastern populations compared to Western cohorts [1,2]. In our experience, this earlier onset appears to be closely linked to the substantial burden of vascular risk factors, such as hypertension and type 2 diabetes, that we observed in almost half of our patients [3]. Our analysis found that older age, abnormal vascular imaging, cardio-aortic embolism, large vessel/cortical stroke, lower HDL cholesterol, and insomnia were independently associated with unfavorable functional outcomes at follow-up. While these outcomes are consistent with international reports, their particular significance in our context underscores the need for risk factor management strategies tailored to Saudi patients [4,5,27].

Sleep disturbances were common in this cohort and appeared clinically relevant, particularly insomnia. In the regression analysis, insomnia was retained as a factor associated with unfavorable functional outcome [28]. However, this association should be interpreted cautiously. Insomnia was identified through routine clinical documentation and patient or caregiver reports rather than through standardized sleep instruments or polysomnography. In addition, insomnia may reflect broader post-stroke complexity, including neurological injury, pain, mood disturbance, psychological stress, reduced mobility, medication effects, or other unmeasured clinical factors. Therefore, our findings do not establish that insomnia directly worsens stroke recovery, but they support further investigation into the role of sleep assessment in post-stroke care. Prospective studies using standardized sleep measures are needed to determine whether identifying and treating insomnia after stroke is associated with improved recovery trajectories [29].

Metabolic markers also contributed to outcome prediction in this cohort. Lower HDL cholesterol was associated with unfavorable functional outcome in the LASSO model, suggesting that lipid-related or broader metabolic health may be relevant to post-stroke recovery [30,31]. However, this finding should not be interpreted as evidence that HDL directly determines functional recovery. HDL may instead serve as a marker of vascular health, metabolic status, inflammation, nutritional status, medication exposure, or other unmeasured factors. Similarly, although LDL cholesterol and total cholesterol ranked highly in the random forest variable-importance analysis, these rankings do not necessarily imply clinical causality. Random forest importance measures can be influenced by correlations among predictors, differences in variable distribution, nonlinear relationships, and interactions between variables. Therefore, the lipid findings should be viewed as hypothesis-generating and should be evaluated further in prospective studies with more complete treatment and longitudinal metabolic data.

The differences between the LASSO and random forest findings also require careful interpretation. LASSO regression identifies variables that retain predictive value within a penalized regression framework, whereas random forest models can capture nonlinear relationships and interactions but provide variable-importance rankings that may be affected by predictor correlation and data structure. In our analysis, LASSO retained insomnia and HDL cholesterol as relevant variables for unfavorable functional outcome, while the random forest model ranked LDL cholesterol and total cholesterol highly. These findings should therefore be considered complementary rather than contradictory. They suggest that sleep and lipid-related variables may contain useful prognostic information, but they do not establish that any individual lipid marker has a direct causal effect on recovery. External validation and prospective analyses are needed before these variables can be incorporated into clinical prediction tools.

For recurrent stroke burden, small artery occlusion and the combined presence of hypertension and type 2 diabetes were associated with multiple documented events. This finding is consistent with the known contribution of chronic vascular risk factors to small vessel disease and recurrent cerebrovascular events [3,13]. However, the association with combined hypertension and diabetes was less robust in penalized regression analysis and should therefore be interpreted cautiously. The observed relationship may reflect cumulative vascular risk, differences in risk-factor control, treatment adherence, follow-up duration, or other unmeasured confounders. The unexpected association between nocturnal awakenings and lower recurrent stroke burden should also be viewed as exploratory. This finding may reflect residual confounding, differences in symptom reporting, health-seeking behavior, or clinical follow-up patterns rather than a protective effect of nocturnal awakenings.

Adding sleep-related information to demographic, vascular, and metabolic variables improved the exploratory prediction of functional outcome compared with a basic clinical model alone. However, these models were developed and evaluated within the same retrospective single-center cohort and have not yet been externally validated [25]. As a result, the random forest findings should be interpreted as exploratory and hypothesis-generating rather than ready for direct clinical application. Future studies should validate these models in larger, independent, and prospectively followed stroke cohorts, ideally with standardized assessment of sleep symptoms, stroke severity, rehabilitation exposure, medication use, and metabolic risk markers.

Several limitations should be acknowledged. First, this was a retrospective single-center study, which limits causal inference and may reduce generalizability to other healthcare settings or patient populations. Second, residual confounding is likely. Important variables that may influence both sleep symptoms and functional outcome were absent, incompletely documented, or not consistently available in the medical records. These include baseline stroke severity, particularly NIHSS score, acute reperfusion therapy, rehabilitation intensity and access, depression and anxiety, sedative or hypnotic medication use, BMI, smoking status, atrial fibrillation, statin use, CPAP treatment, and follow-up duration. Each of these factors may affect sleep quality, vascular risk, recurrent stroke, and functional recovery. Third, sleep disturbances were identified from routine clinical documentation rather than standardized questionnaires, actigraphy, or polysomnography, increasing the possibility of misclassification and underreporting. Fourth, treatment exposure and adherence after discharge were not fully captured, including lipid-lowering therapy, antihypertensive control, diabetes management, and sleep-specific interventions. Finally, the predictive models were internally evaluated but not externally validated, and variable-importance rankings from machine learning models should be interpreted cautiously. These limitations mean that our findings should be considered hypothesis-generating and require confirmation in prospective, multicenter studies with standardized sleep, metabolic, treatment, and functional outcome assessments.

## 5. Conclusions

In conclusion, this retrospective single-center cohort study found that older age, adverse vascular characteristics, documented insomnia, and lower HDL cholesterol were associated with unfavorable functional outcome after stroke, whereas recurrent stroke events were associated mainly with small artery occlusion and combined hypertension plus type 2 diabetes. These findings should be interpreted as observational associations rather than causal effects. The results suggest that sleep-related symptoms and metabolic markers may help identify patients at higher risk of poor post-stroke outcomes, but they require confirmation in prospective, multicenter studies using standardized follow-up and objective sleep assessment.

## Figures and Tables

**Table 1:** Baseline clinical, sleep-related, and laboratory characteristics overall and by functional outcome.

Variable	Overall (N = 270)	Favorable (N = 214)	Unfavorable (N = 56)	*p*-Value
Age, mean (SD), years	54.5 (13.1)	53.4 (12.1)	58.7 (15.6)	0.021
Male sex, n (%)	164 (60.7)	127 (59.3)	37 (66.1)	0.445
Final diagnosis: stroke, n (%)	264 (97.8)	208 (97.2)	56 (100.0)	0.448
Hemorrhage, n (%)	11 (4.1)	7 (3.3)	4 (7.1)	0.355
Territory infarction, n (%) *				0.011
Large vessel/cortical stroke	152 (56.3)	113 (52.8)	39 (69.6)	
No stroke/unspecified	65 (24.1)	60 (28.0)	5 (8.9)	
Small vessel stroke	52 (19.3)	40 (18.7)	12 (21.4)	
Vascular imaging, n (%)				<0.001
Normal/no significant finding	203 (75.2)	173 (80.8)	30 (53.6)	
Steno-occlusive/atherosclerotic disease	36 (13.3)	21 (9.8)	15 (26.8)	
Vessel territory/occlusion pattern	31 (11.5)	20 (9.3)	11 (19.6)	
Subclinical, yes, n (%)	121 (44.8)	98 (45.8)	23 (41.1)	0.63
Large artery disease, yes, n (%)	48 (17.8)	33 (15.4)	15 (26.8)	0.074
Cardio-aortic embolism, yes, n (%)	48 (17.8)	30 (14.0)	18 (32.1)	0.003
Small artery occlusion, yes, n (%)	58 (21.5)	45 (21.0)	13 (23.2)	0.864
Undetermined causes, yes, n (%) *	44 (16.3)	34 (15.9)	10 (17.9)	0.901
Hypertension/type 2 diabetes risk profile, n (%)				0.156
None	56 (20.7)	41 (19.2)	15 (26.8)	
Hypertension	41 (15.2)	35 (16.4)	6 (10.7)	
T2D	40 (14.8)	28 (13.1)	12 (21.4)	
Hypertension + T2D	133 (49.3)	110 (51.4)	23 (41.1)	
Insomnia, yes, n (%)	64 (23.7)	40 (18.7)	24 (42.9)	<0.001
Circadian rhythm disturbance, yes, n (%)	78 (28.9)	58 (27.1)	20 (35.7)	0.271
Nocturnal awakenings, yes, n (%)	161 (59.6)	126 (58.9)	35 (62.5)	0.735
Sleep apnea risk, yes, n (%)	120 (44.4)	89 (41.6)	31 (55.4)	0.09
Number of stroke events, mean (SD)	1.14 (0.57)	1.12 (0.52)	1.23 (0.74)	0.295
Sleep latency, mean (SD), min	32.5 (16.9)	31.6 (15.7)	36.1 (20.7)	0.137
Nap frequency, mean (SD)	1.44 (1.26)	1.42 (1.25)	1.55 (1.32)	0.484
Total cholesterol, mean (SD), mmol/L *	4.66 (1.26)	4.68 (1.25)	4.61 (1.33)	0.727
HDL cholesterol, mean (SD), mmol/L *	1.16 (0.35)	1.18 (0.35)	1.07 (0.32)	0.034
LDL cholesterol, mean (SD), mmol/L *	3.01 (1.16)	3.01 (1.10)	3.04 (1.36)	0.868
Triglycerides, mean (SD), mmol/L *	1.57 (0.86)	1.56 (0.83)	1.57 (0.96)	0.952
Glucose, mean (SD) #	163 (87.5)	159 (75.4)	175 (119.0)	0.482

Data are presented as n (%) for categorical variables and mean (SD) for continuous variables. *p*-values compare patients with favorable versus unfavorable functional outcomes. Functional outcome was defined as favorable (mRS 0–2) or unfavorable (mRS 3–6). * A small number of values were missing for territory infarction (N = 1), undetermined causes (N = 2), total cholesterol (N = 8), HDL cholesterol (N = 7), LDL cholesterol (N = 8), and triglycerides (N = 4). # EEG/ECG and glucose variables had substantial missingness and should be interpreted with caution. Missingness for EEG/ECG was 82.2% overall (87.9% in the favorable group and 60.7% in the unfavorable group), and missingness for glucose was 49.6% overall (51.4% in the favorable group and 42.9% in the unfavorable group).

**Table 2:** Baseline clinical, sleep-related, and laboratory characteristics overall and by recurrent stroke event.

Variable	Overall (N = 270)	0–1 Documented Event (N = 246)	Recurrent Events (N = 24)	*p*-Value
Age, mean (SD), years	54.5 (13.1)	54.4 (12.6)	55.2 (17.8)	0.845
Male sex, n (%)	164 (60.7)	149 (60.6)	15 (62.5)	1
Final diagnosis: stroke, n (%)	264 (97.8)	240 (97.6)	24 (100.0)	0.961
Hemorrhage, n (%)	11 (4.1)	9 (3.7)	2 (8.3)	0.572
Territory infarction, n (%) *				0.63
Large vessel/cortical stroke	152 (56.3)	138 (56.1)	14 (58.3)	
No stroke/unspecified	65 (24.1)	58 (23.6)	7 (29.2)	
Small vessel stroke	52 (19.3)	49 (19.9)	3 (12.5)	
Vascular imaging, n (%)				0.621
Normal/no significant finding	203 (75.2)	183 (74.4)	20 (83.3)	
Steno-occlusive/atherosclerotic disease	36 (13.3)	34 (13.8)	2 (8.3)	
Vessel territory/occlusion pattern	31 (11.5)	29 (11.8)	2 (8.3)	
Subclinical, yes, n (%)	121 (44.8)	117 (47.6)	4 (16.7)	0.007
Large artery disease, yes, n (%)	48 (17.8)	43 (17.5)	5 (20.8)	0.896
Cardio-aortic embolism, yes, n (%)	48 (17.8)	41 (16.7)	7 (29.2)	0.212
Small artery occlusion, yes, n (%)	58 (21.5)	45 (18.3)	13 (54.2)	<0.001
Undetermined causes, yes, n (%) *	44 (16.3)	40 (16.3)	4 (16.7)	1
Hypertension/type 2 diabetes risk profile, n (%)				0.006
None	56 (20.7)	54 (22.0)	2 (8.3)	
Hypertension	41 (15.2)	40 (16.3)	1 (4.2)	
T2D	40 (14.8)	39 (15.9)	1 (4.2)	
Hypertension + T2D	133 (49.3)	113 (45.9)	20 (83.3)	
Insomnia, yes, n (%)	64 (23.7)	57 (23.2)	7 (29.2)	0.683
Circadian rhythm disturbance, yes, n (%)	78 (28.9)	71 (28.9)	7 (29.2)	1
Nocturnal awakenings, yes, n (%)	161 (59.6)	152 (61.8)	9 (37.5)	0.036
Sleep apnea risk, yes, n (%)	120 (44.4)	110 (44.7)	10 (41.7)	0.943
Sleep latency, mean (SD), min	32.5 (16.9)	32.8 (17.3)	29.8 (12.4)	0.282
Nap frequency, mean (SD)	1.44 (1.26)	1.43 (1.26)	1.58 (1.28)	0.582
Total cholesterol, mean (SD), mmol/L *	4.66 (1.26)	4.70 (1.28)	4.31 (1.00)	0.092
HDL cholesterol, mean (SD), mmol/L *	1.16 (0.35)	1.17 (0.34)	1.05 (0.38)	0.161
LDL cholesterol, mean (SD), mmol/L *	3.01 (1.16)	3.04 (1.17)	2.71 (0.96)	0.129
Triglycerides, mean (SD), mmol/L *	1.57 (0.86)	1.55 (0.86)	1.72 (0.84)	0.371
Glucose, mean (SD) #	163 (87.5)	160 (84.1)	179 (108.0)	0.479

* Lipid measures and some etiologic classifications had small amounts of missing data. # EEG/ECG and glucose had substantial missing data.

## Data Availability

The raw data supporting the conclusions of this article will be made available by the corresponding author on request.

## Supplementary Materials

The following supporting information can be downloaded at https://www.mdpi.com/article/10.3390/jcm15145394/s1: Table S1. Baseline clinical, and sleep-related characteristics overall and by functional outcome; Table S2. Baseline clinical and sleep-related characteristics overall and by recurrent stroke events; Table S3. Penalized logistic regression coefficients and odds ratios for predictors of unfavorable functional outcome.

## Abbreviations

The following abbreviations are used in this manuscript:

KFHU	King Fahd Hospital of the University
mRS	Modified Rankin Scale
T2D	Type 2 Diabetes
HDL	High-density lipoprotein
LDL	Low-density lipoprotein
EEG	Electroencephalogram
ECG	Electrocardiogram
AUC	Area under the curve
