# Supplementary material for: Sleep Disturbances, Metabolic Markers, and Outcomes After Stroke: A Retrospective Cohort Study in a Tertiary Hospital
Source: J Clin Med. 2026 Jul 9;15(14):5394. doi: 10.3390/jcm15145394 (PMC13410559; doi:10.3390/jcm15145394)
Supplement: Supplementary file 1 [file jcm-15-05394-s001.zip › jcm-4404211-supplementary.pdf]

Supplementary Tables

Supplementary Table S1. Baseline clinical, and sleep-related characteristics overall and by functional outcome

Sleep apnea risk, yes, n (%)	120 (44.4)	89 (41.6)	31 (55.4)	0.09
EEG/ECG, n (%)[#]				**0.002**
Normal	14 (5.2)	13 (6.1)	1 (1.8)	
Abnormal	34 (12.6)	13 (6.1)	21 (37.5)	
Aspirin use, n (%)	263 (97.4)	209 (97.7)	54 (96.4)	0.964
Nap duration, mean (SD), min	19.4 (16.1)	19.2 (16.5)	20.1 (14.7)	0.682

Supplementary Table S2. Baseline clinical and sleep-related characteristics overall and by recurrent stroke events

Sleep apnea risk, yes, n (%)	120 (44.4)	110 (44.7)	10 (41.7)	0.943
EEG/ECG, n (%)#				0.216
Normal	14 (5.2)	9 (3.7)	5 (20.8)	
Abnormal	34 (12.6)	29 (11.8)	5 (20.8)	
Aspirin use, n (%)	263 (97.4)	239 (97.2)	24 (100.0)	0.869
Nap duration, mean (SD), min	19.4 (16.1)	19.6 (16.4)	16.7 (12.9)	0.307

Supplementary table S3. Penalized logistic regression coefficients and odds ratios for predictors of unfavorable functional outcome.

base_var	Variable_label2	Coefficient	CI_low	CI_high	OR	OR_low	OR_high
vascular imaging	Vascular Imaging (Steno-occlusive / Atherosclerotic Disease vs. Normal)	1.056399	0.71922	1.370281	2.876	2.053	3.936
cardio_aortic_embolism	Cardio-Aortic Embolism (Yes vs. No)	0.847118	0.581851	1.0936	2.333	1.789	2.985
insomnia	Insomnia (Yes vs. No)	0.673935	0.425543	0.908421	1.962	1.53	2.48
vascular_imaging	Vascular Imaging (Vessel Territory / Occlusion Pattern vs. Normal)	0.371595	0	0.686728	1.45	1	1.987
territory_infarction	Territory of Infarction (Large Vessel / Cortical Stroke vs. No Stroke / Unspecified)	0.147174	0	0.371289	1.159	1	1.45
circadian_rhythm	Circadian Rhythm Disruptions (Yes vs. No)	0.133393	0	0.352151	1.143	1	1.422
uncommon_causesT2D	Uncommon Causes (T2D vs. None)	0.128911	0	0.435085	1.138	1	1.545
large_artery	Large Artery Atherosclerosis (Yes vs. No)	0.058594	0	0.34119	1.06	1	1.407
hemorrhage	Hemorrhage (Yes vs. No)	0.018159	0	0.543358	1.018	1	1.722
sleep_apnea_risk	Sleep Apnea Risk (Yes vs. No)	0.016064	0	0.241651	1.016	1	1.273
age	Age (Years)	0.011104	0.001478	0.01914	1.011	1.001	1.019
Hypertension/type 2 diabetes risk profile	Uncommon Causes (Hypertension & T2D vs. None)	-0.05879	-0.29797	0	0.943	0.742	1
hdl_mmol	HDL Cholesterol (mmol/L)	-0.4901	-0.78308	-0.18561	0.613	0.457	0.831
